# Inconsistency Calibrating Algorithms for Large Scale Piezoresistive Electronic Skin

**DOI:** 10.3390/mi11020162

**Published:** 2020-02-03

**Authors:** Jinhua Ye, Zhengkang Lin, Jinyan You, Shuheng Huang, Haibin Wu

**Affiliations:** School of Mechanical Engineering and Automation, Fuzhou University, Fuzhou 350116, China; yejinhua@fzu.edu.cn (J.Y.); linzksmile@gmail.com (Z.L.); youjinyanyan@163.com (J.Y.); shuhenghuang.cn@gmail.com (S.H.)

**Keywords:** calibration, electronic skin, inconsistency, large scale, piezoresistive

## Abstract

In the field of safety and communication of human-robot interaction (HRI), using large-scale electronic skin will be the tendency in the future. The force-sensitive piezoresistive material is the key for piezoresistive electronic skin. In this paper, a non-array large scale piezoresistive tactile sensor and its corresponding calibration methods were presented. Because of the creep inconsistency of large scale piezoresistive material, a creep tracking compensation method based on K-means clustering and fuzzy pattern recognition was proposed to improve the detection accuracy. With the compensated data, the inconsistency and nonlinearity of the sensor was calibrated. The calibration process was divided into two parts. The hierarchical clustering algorithm was utilized firstly to classify and fuse piezoresistive property of different regions over the whole sensor. Then, combining the position information, the force detection model was constructed by Back-Propagation (BP) neural network. At last, a novel flexible tactile sensor for detecting contact position and force was designed as an example and tested after being calibrated. The experimental results showed that the calibration methods proposed were effective in detecting force, and the detection accuracy was improved.

## 1. Introduction

Tactility is an essential perception for intelligent robots to acquire external information [1]. It plays an important role in safe human-robot interaction [2,3]. Electronic skin can simulate the human tactile nervous system and sense information such as pressure [4], position [5], temperature [6], and texture [7] and so on for subsequent motion control in real time. With the growing demand of whole-body sensing, people have begun to focus on the study of large-scale electronic skin which is often called tactile sensor. A series of research on tactile sensing technology have already been carried out. It has made great breakthroughs in piezoresistive tactile sensors, whose structure can be divided into array and non-array style.

By splicing small pieces of high precision sensing units or integrating them into a large substrate in a certain order, array sensors can achieve large area tactile detection. Luo et al. [8] presented a flexible 6 × 6 pressure-sensitive array based on the porous substrate. The graphene-coated foams (GCF) were integrated in it as sensing units for pressure perception. Wang et al. [9] designed a novel skin-like tactile sensor array to measure the contact pressure of curved surfaces. It used nickel powder filled polymer and Silver-Nanowires/Polydimethylsiloxane (AgNWs/PDMS) forming stretchable interconnects. Cheng et al. [10] proposed an approach to realize a large-area highly-twistable artificial skin, and each tactile sensing element was formed by attaching conductive polymer on the spiral electrodes. The sensor can detect force through the relationship of electrical resistance and pressure on every tactile sensing element.

However, the detection area of array sensors is incontinuous, and the sensing information is acquired by unit-to-unit scanning. By comparison, non-array tactile sensors are integrative in structure without detection blind spots. It doesn’t need flexible complicated array electrodes, and is easy to be fabricated. That’s why non-array structure is more suitable for large area force detection. Yao, A., et al. [11] designed an based Electrical Impedance Tomography fabric sensor that can provide a pressure map using some current carrying and voltage sensing electrodes attached to the boundary of the fabric patch. Lee et al. [12] proposed a multi-point and multi-directional strain mapping sensor based on multiwall carbon nanotube (MWCNT)-silicone elastomer nanocomposites and anisotropic electrical impedance tomography (aEIT). It can successfully estimate surface normal forces. For these sensors, the amount of data to be collected and calculated in each detecting cycle is large, which requires a higher detection system to improve real-time performance. Moreover, the imaging algorithms are relatively complicated.

Although piezoresistive composite is a good force-sensing element for flexible tactile sensor, it is viscoelastic and has great time-dependent character, i.e., the creep. It influences precision of sensors seriously and must be improved before sensor calibration. At present, there are many studies on creep property of the composites. The numerous viscoelastic creep models [13] have been constructed through experimental analysis, but few studies on how to compensate the creep. In general, there are two ways to solve the problem. On the one hand, creep can be reduced through the selection of matrix materials and manufacturing process. Huang, Y., et al. [14] found the creep resistance of the conductive rubber composites can be improved by doping certain nano-SiO_2_. Wang, P., et al. [15] studied the variation of electrical resistance under uniaxial pressure for carbon black-silicone rubber composite, and developed a novel way to mitigate resistance creep in rubber composites by the inclusion of white silica fillers. But not every piezoresistive material could match a suitable doping dose material to alleviate the creep character. Even the method of doping has been adopted, it is difficult to thoroughly eliminate the creep. On the other hand, we can compensate the creep through the signal processing algorithm or electronic circuit of the sensors. Ahmed, M.M. et al. [16] built a Levenberg-Marquard BackPropagation (LMBP) neural network model of self-calibration for a pressure sensor, which successfully predicted the desired pressure over time and reduced the impact of creep. Wang, L., et al. [17] used a linear combination of two negative exponential functions to fit the data of creep. Kim [18] described the creep as a superposition of exponential functions. Because of difference of creep character under different contact conditions and regions, it is hard to be compensated by fitting a unified function model.

In this paper, we presented the creep compensation method to ensure the accuracy of data used for calibration. Then, the calibration method was used to alleviate inconsistency and nonlinearity of large scale piezoresistive tactile sensors. Combining with our previous researches, a novel large scale tactile sensor for detecting contact position and force was designed as a test example to verify these methods.

## 2. Principle of the Large Scale Piezoresistive Tactile Sensor

**Figure 1:**
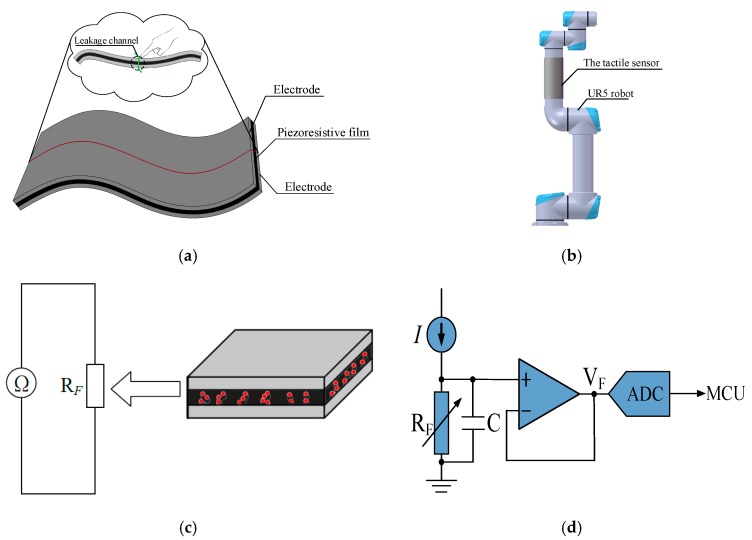
The structure and detection principle of the tactile sensor: (**a**) The sandwich structure; (**b**) The sensor covered on UR5 robot; (**c**) The equivalent resistance in the pressed position; (**d**) The contact force detection circuit.

The piezoresistive film is mostly used in tactile sensor for contact force detection. In this paper, a large scale of piezoresistive film as a whole non-array structure is adopted to design a sensor shown in Figure 1a. The sensor can be easily attached to the robot surface, as shown in Figure 1b. It has three layers including upper electrode layer, piezoresistive film layer and lower electrode layer, and each of them closes to each other tightly. The electrode layers are both good conductors. When anywhere of the sensor is pressed, the resistance between the upper and lower electrode layer at the contact position of the piezoresistive film will decrease. It is just like an electric leakage channel at the pressed position when an excitation is applied to the two electrodes, but the resistance of the unpressed region remains large. With the characteristics of piezoresistive film, there is a negative correlation between the applied pressure and the equivalent resistance. Therefore, the resistance change of the piezoresistive film between the upper and lower electrode layers can reflect the external force.

For the piezoresistive tactile sensor as shown in Figure 1a, the equivalent resistance $R_{F}$ between upper and lower electrode layer can be measured using a detection circuit. As long as the resistance of the film under external pressure is measured, it is possible to deduce the pressure F depending on the relationship of F and $R_{F}$.

The detection circuit of the contact force can realize the conversion from resistance to voltage by a simple circuit, as shown in Figure 1d. The sensor is connected with a constant current source (CCS). The CCS can provide constant current. The voltage at the connection point is buffered by a voltage follower, and can be transmitted to the microcontroller for the A/D conversion. Finally, the output resistance $R_{F}$ can be calculated by Equation (1).

(1) $$V_{F}=I\cdot R_{F}$$

**Figure 2:**
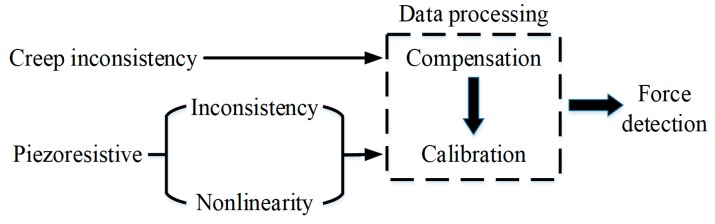
The sensor data processing.

But, the piezoresistive film is normally fabricated by mixing some conductive particles into a viscoelastic insulating substrate. This kind of piezoresistive film has the creep characteristics, and the creep is inconsistent for different region over the whole film. The piezoresistive characteristics of the film are also nonlinear and inconsistent. In order to get consistent force measurements over the full film, the problems of creep inconsistency, piezoresistive inconsistency and nonlinearity should be resolved. A new data processing algorithm was proposed including creep compensation and force calibration as shown in Figure 2. The whole procession includes two algorithms. One is creep compensation algorithm, and the other is force calibration algorithm calibrating for piezoresistive inconsistence and nonlinearity. The creep of the sensor should be compensated before the subsequent calibration.

## 3. Creep Compensation before Sensor Calibration

### 3.1. Creep Process Analysis

**Figure 3:**
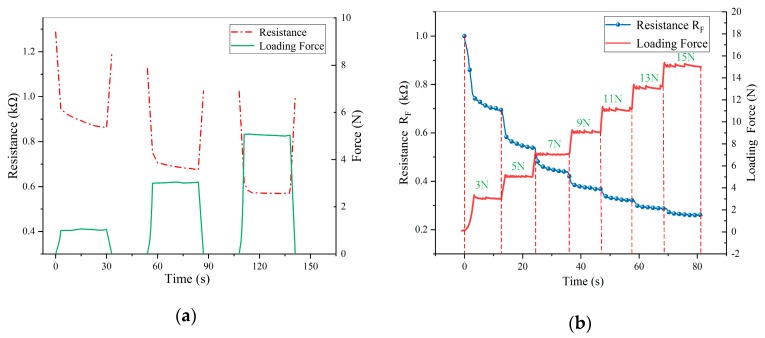
Creep of the sensor under different force imposing situations: (**a**) Imposing different force independent; (**b**) Imposing force incremental step by step.

It’s known that the piezoresistive film exists creep and creep inconsistency. Two tests were conducted on the sensor. One is that 1 N force was pressed at a position, and was unloaded after keeping about 30 s. After a while, 3 N, 5 N of force were pressed at the same position in sequence. The creep characteristic was shown in Figure 3a. The other is that an incremental stepper force was pressed at a position of the sensor, as shown in Figure 3b.

**Figure 4:**
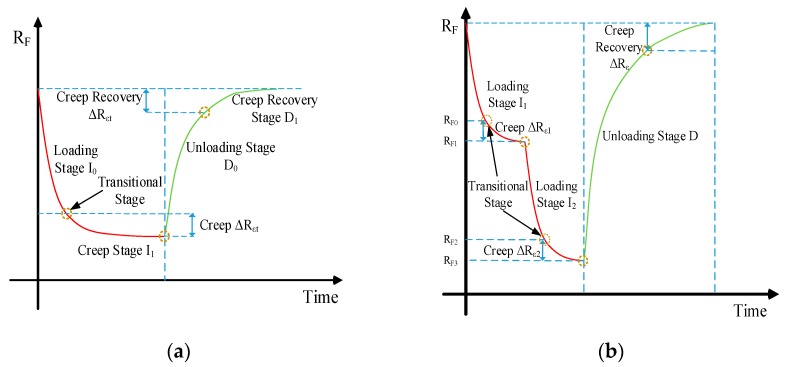
The sensor output resistance under different working stages: (**a**) Loading force once; (**b**) Loading force many times.

As shown in Figure 3, the resistance is still getting smaller under a constant force, but the downward trend slows down as time goes by. As the pressure increases, the resistance variation caused by creep also decreases. In summary, the working process of the sensor can be divided into three stages, as shown in Figure 4a,b.

(1) Loading/unloading stage: the resistance increases or decreases significantly.
(2) Creep stage: when the loading force remains constant, the resistance decreases slowly.
(3) Transitional stage: it closes to the junction of loading/unloading and creep stage, and represented by the circle in the following figure.

**Figure 5:**
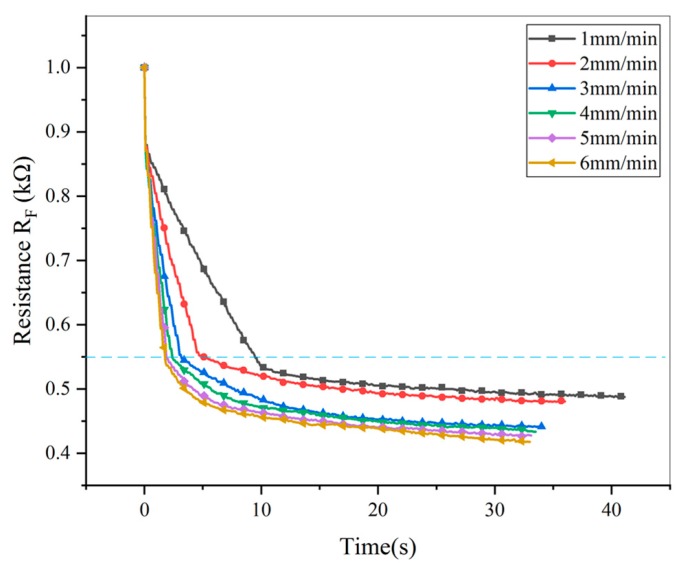
Sensor output resistance at different loading speed.

Actually, once the sensor is compressed, the creep occurs. To study the creep of the sensor in loading stage, the central position of the sensor was compressed for a test. A maximal 3 N force was applied to the sensor at different loading speed of 1, 2, 3, 4, 5 and 6 mm/min respectively. When loading to 3 N, the force was maintained for 30 s. The results were shown in Figure 5.

Curves above the dotted line in Figure 5 is the loading stage, and curves below the dotted line is the creep stage. In the case of different loading speeds, the resistances at the full load point are still approximately equal. That means the creep during loading stage can be neglected. After entering into creep stage, the faster the loading speed is, the more obvious the creep is. We have to compensate creep after loading stage

In summary, the sensor is difficult to provide accurate force feedback, and using a simple function fitting method can’t cope with such different contact situations. A new creep tracking compensation method was proposed to compensate the creep. firstly, the resistance variation during the creep stage was sampled, based on the known critical point between loading and creep stage. Then, the resistance corresponding to the actual contact force can be obtained by subtracting creep-induced resistance variations. The specific formula is as follows:

(2) $$\{\begin{array}{l} R_{f_{load/unload}}^{\left(n\right)}=R_{ft}^{\left(n\right)}-\Delta R_{\varepsilon }^{\left(n-1\right)}\\ R_{f_{creep}}^{\left(n\right)}=R_{ft}^{\left(n\right)}-\Delta R_{\varepsilon }^{\left(n-1\right)}-\Delta R_{\varepsilon t^{\prime }}^{\left(n\right)} \end{array}$$

where n is the number of contact force change. $R_{f_{load/unload}}^{\left(n\right)}$ and $R_{f_{creep}}^{\left(n\right)}$ are the sensor output resistances corresponding to actual contact force in the loading/unloading and creep stage, respectively. $R_{ft}^{\left(n\right)}$ is the resistance at time t. $\Delta R_{\varepsilon t^{\prime }}^{\left(n\right)}$ is the resistance variation caused by the creep at time $t^{\prime }$ of n-th creep stage. $\Delta R_{\varepsilon }^{\left(n-1\right)}$ is the resistance variation caused by creep accumulation during previous (n−1) creep stages.

According to Equation (2), it is important to determine whether the current working stage of the sensor is in loading stage or creep stage. As shown in Figure 5, the output resistance curve is steep on the left of the critical point, but gentle on the right. Relative variation of the resistance is used to describe degree of change and expressed as follows:

(3) $$\delta =\frac{\left|\Delta R_{f1}\right|}{R_{f1}}=\frac{\left|R_{f1}-R_{f0}\right|}{R_{f1}}$$

where $R_{f0}$ is the sensor output resistance at the previous period. $R_{f1}$ is the sensor output resistance at the current period.

**Figure 6:**
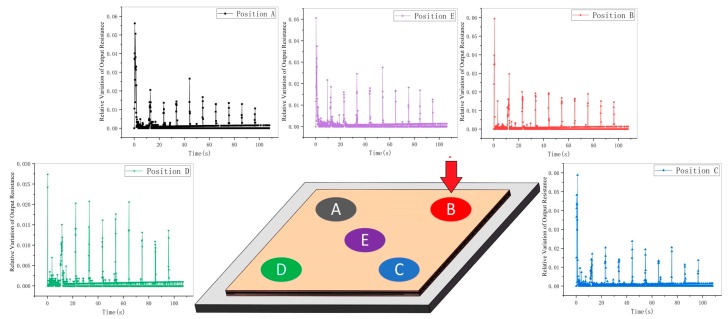
Relative variation of sensor output resistance at different positions.

The creep at different positions and forces was further studied by relative variation. Four corners (ABCD) and the central position (E) of the sensor were chosen as tested areas. An incremental stepper force was applied to them from 1 N to 10 N (at an interval of 1 N). After each change, the force was kept for 10 s. We collected the sensor output resistance and calculated the relative variation by Equation (3). The results were shown in Figure 6.

Based on the analysis of the Figure 6, the relative variations of resistance in the loading stage and creep stage are obviously different. The peaks belong to loading stage, and the troughs belong to creep stage. In other word, the resistance changes more greatly in the loading stage. It is an important basis for determining the working state of the sensor.

### 3.2. Creep Tracking Compensation

In this paper, the sample data in Figure 6 were used as a data space. The K-means clustering algorithm was adopted to find out the boundaries among different working stages of sensor. The specific algorithm is as follows:

(1) The pressing process of the sensor can be divided into loading stage, transitional stage and creep stage, so 3 set of sample data in the data space were selected as the initial cluster centers.

(2) The absolute distance between the remaining sample data and the current three cluster centers were calculated. According to the K-means clustering algorithm, the nearest distance criterion is defined. Because the samples clustered in this paper is one-dimensional data and the type of samples data is resistance value, we use the absolute distance which is simplest and most direct to define the nearest distance criterion. These sample data were classified into corresponding categories and can be expressed as:

(4) $$C_{k}=\left\{n:k=\arg\underset{k}{\min}\left|\delta_{n}-\mu_{k}\right|\right\}$$

where $C_{k}$ is the set of sample date belonging to cluster k. $\mu_{k}$ is the cluster center of cluster *k*.

(3) The averages of all sample data in each category were calculated and taken as the new cluster centers of the category. The update of cluster centers can be expressed as:

(5) $$\mu_{k}=\frac{1}{N_{k}}\sum_{n\in C_{k}}\delta_{n}$$

(4) The sum of weighted mean absolute distance $G_{J}$ used as clustering criterion functions can be expressed as:

(6) $$\{\begin{array}{l} G_{J}=\sum_{k=1}^{3}p_{k}s_{k}^{*}\\ s_{k}^{*}=\frac{2}{N_{k}\left(N_{k}-1\right)}\sum_{i\in C_{k}}\sum_{j\in C_{k}}\left|\delta_{i}-\delta_{j}\right|\\ p_{k}=\frac{N_{k}}{N} \end{array}$$

where $p_{k}$ is the priori probability, which can be calculated by the total number of sample data N and the number of sample data $N_{k}$ of each category. $s_{k}^{*}$ is the average of absolute distance between sample data of cluster k. After updating the cluster centers each time, $G_{J}$ was calculated. When $G_{J}$ converged to a minimum value or cluster centers no longer changed, it was judged as the end of clustering. Then, the final clustering center and boundary can be obtained. Otherwise, return to step (2).

**Figure 7:**
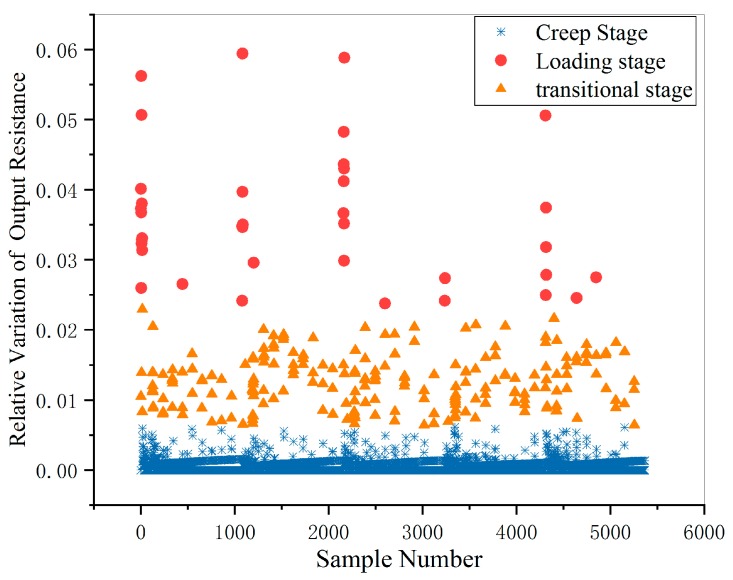
The results of K-means clustering.

After K-means clustering, the results were shown in Figure 7. The boundaries of loading stage, creep stage and transitional stage can be seen clearly.

To verify the rationality of clustering results, the silhouette coefficient was introduced as the evaluation index of cluster density and dispersion. It can be calculated by the following formula:

(7) $$\{\begin{array}{l} s\left(i\right)=\frac{a\left(i\right)-b\left(i\right)}{\max\left\{a\left(i\right),b\left(i\right)\right\}}\\ S=\frac{\sum_{i=1}^{N}s\left(i\right)}{N} \end{array}$$

Where a(i) is the average of the distance between sample i and the other sample of the same cluster. b(i) is the minimum of the average distance between sample i and the samples in other categories. The range of silhouette coefficient is from −1 to 1. The closer its value is to 1, the more reasonable the clustering is.

**Figure 8:**
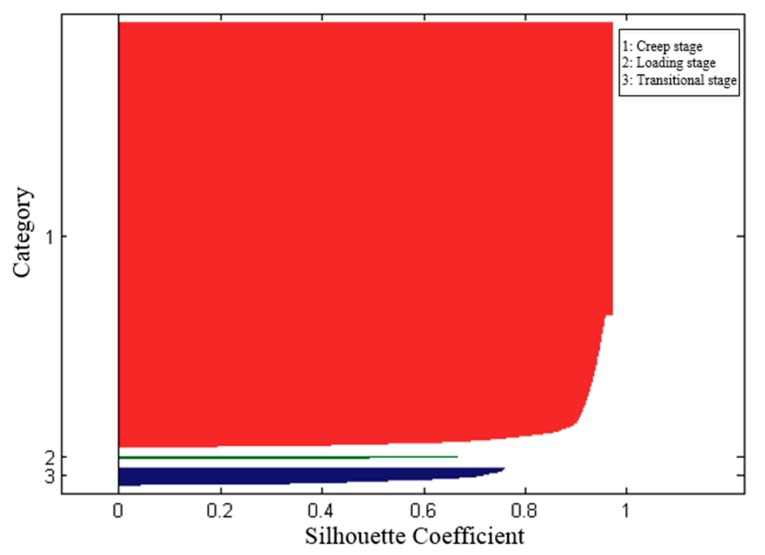
The silhouette of categories.

The silhouette of each category was shown in Figure 8. The silhouette coefficient of the final clustering results is 0.931 by Equation (7). That is, the clustering in this paper is reasonable.

Due to the fuzziness of transitional stage, it is difficult to identify the critical point accurately between loading and creep stages. In this paper, the concept of fuzzy mathematics was introduced to solve it. The relative variation of resistance was taken as the discourse domain V. $T_{1}$ and $T_{2}$ were the fuzzy subsets({loading stage} and {creep stage}) of V. Based on the boundary of different categories obtained from the clustering result, the membership functions of loading stage and creep stage can be defined by fuzzy distribution of semi-trapezoidal type as follows:

(8) $$T_{1}\left(\delta \right)=\{\begin{array}{cc} 0 & \delta \leq 0.00612\\ \frac{\delta -0.00612}{0.01762} & 0.00612<\delta \leq 0.02374\\ 1 & \delta >0.02374 \end{array}$$

(9) $$T_{2}\left(\delta \right)=\{\begin{array}{cc} 0 & \delta >0.02374\\ \frac{0.02374-\delta }{0.01762} & 0.00612<\delta \leq 0.02374\\ 1 & \delta \leq 0.00612 \end{array}$$

With the help of the maximum membership principle, the current working stage of the sensor was recognized by fuzzy recognition. Comparing $T_{1}\left(\delta \right)$ with $T_{2}\left(\delta \right)$ at the current moment, if $T_{1}\left(\delta \right)$ is greater than $T_{2}\left(\delta \right)$, the sensor is in loading stage. Otherwise, it is in creep stage. Then, the formula Equation (2) of creep compensation was chosen according to the current working state of sensor.

Four experiments were used to verify the feasibility of the method as follows:

(1) An incremental stepper force of 1 N to 7 N (at 1 N intervals) was applied to three random positions of the sensor. After each increase, the force was maintained for 10 s.
(2) The sensor central position was selected to be applied a force of 6 N firstly at a loading speed of a. When reaching to 6 N, the force remained constant for 5 s. Then, the force was increased continuously to 9 N at a loading speed of b, and remained it for a period of time.
(3) 5 N single loading was applied to a random position of the sensor.
(4) An incremental stepper force of 1 N to 10 N (at 1 N intervals) was applied to the position which is same as the position of experiment (3). After each increase, the force was maintained for 10 s.

**Figure 9:**
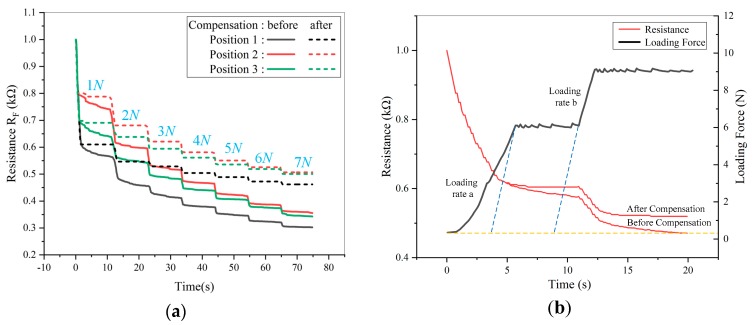
The sensor output resistance after creep compensation: (**a**) Different positions; (**b**) Different loading speeds.

**Figure 10:**
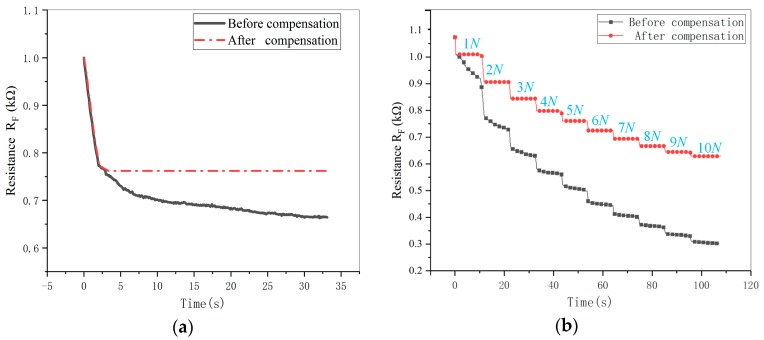
Results of creep compensation at the same position: (**a**) Result of 5 N single loading creep compensation; (**b**) Result of multiple loading creep compensation.

The results of the first two experiments (Experiment (1), Experiment (2)) were shown in Figure 9a,b. The results of the last two experiments (Experiment (3), Experiment (4)) were shown in Figure 10a,b.

As shown in Figure 9 and Figure 10, after K-means clustering analysis and defining appropriate membership function, the sensor working stage can be identified effectively. Meanwhile, the creep was also compensated by Equation (2), no matter what force, position and loading speed were. In particular, it ensures the accuracy and stability of the collected sensor data for force calibration to a certain extent.

## 4. Fusion and Calibration for Piezoresistive Units

### 4.1. Clustering Analysis of Sensing Units

Because of the piezoresistive inconsistency over the full film, when calculating the force loaded on the sensor, it is not enough to only consider the mapping between the force and the sensor output resistance. The different pressed position must be taken into account. In this paper, the whole sensor was regarded as a combination of several units, and the mapping relationship of each unit between the force and the resistance is established. Because the data detected in different units have a certain degree of redundancy and the boundaries between different sensing units have force detection error which is caused by positional error (the maximum positional error is 3 mm in the paper), before fitting the mathematical model, it is necessary to cluster and fuse the data after creep compensation for different positions. It can classify the positions with similar piezoresistive properties into the same categories, so as to reduce the computational complexity of subsequent fitting and reduce error of borders by reducing number of borders.

However, it is hard to determine where the piezoresistive characteristics of the sensor are similar at first. This paper used agglomerative nesting (AGNES) algorithm to find how many sensing units should be divided. The specific algorithm is as follows:

(1) The sensor was evenly divided into N independent sensing units, and a force of 1 N~15 N, at intervals of 1 N, was applied to them respectively. The obtained output resistances of the units were used as initial categories. They can be expressed as follows:

(10) $$\begin{array}{cc} R_{Fi}=\left[r_{i1};r_{i2};r_{i3};...;r_{i15}\right] & \left(i=1,2,3,...,N\right) \end{array}$$

$d_{ij}^{2}$ is defined as the Euclidean square distance of sample i and j. The formula is as follows:

(11) $$d_{ij}^{2}=\sum_{k=1}^{15}{\left(R_{Fik}-R_{Fjk}\right)}^{2}$$

(2) The distance between two clusters of the initial clusters was calculated to obtain the initial distance matrix, and Ward’s method was used as the basis of clustering in this paper. To describe similarity of piezoresistive characteristics of two sensing units, the distance between clusters was defined as follows:

(12) $$Dist\left(p,q\right)=\frac{n_{p}n_{q}}{n_{p}+n_{q}}d_{\bar{p}\bar{q}}^{2}$$

where $n_{p}$ and $n_{q}$ is the number of samples of cluster p and q respectively. $\bar{p}$ and $\bar{q}$ are the centroids of cluster p and q respectively.

(3) The two clusters with the smallest distance between clusters were merged. For example, if the distance between cluster p and cluster q is the smallest, they can be merged as a new cluster k for the basis of the next clustering. The distance between cluster k and the rest cluster r can be calculated by the following formula:

(13) $$Dist\left(k,r\right)=\frac{n_{p}+n_{r}}{n_{k}+n_{r}}Dist\left(p,r\right)+\frac{n_{q}+n_{r}}{n_{k}+n_{r}}Dist\left(q,r\right)-\frac{n_{r}}{n_{k}+n_{r}}Dist\left(p,q\right)$$

where $n_{k}$ and $n_{r}$ is the number of samples of cluster k and r respectively.

(4) Step (3) was repeated until all categories were merged into a category.

**Figure 11:**
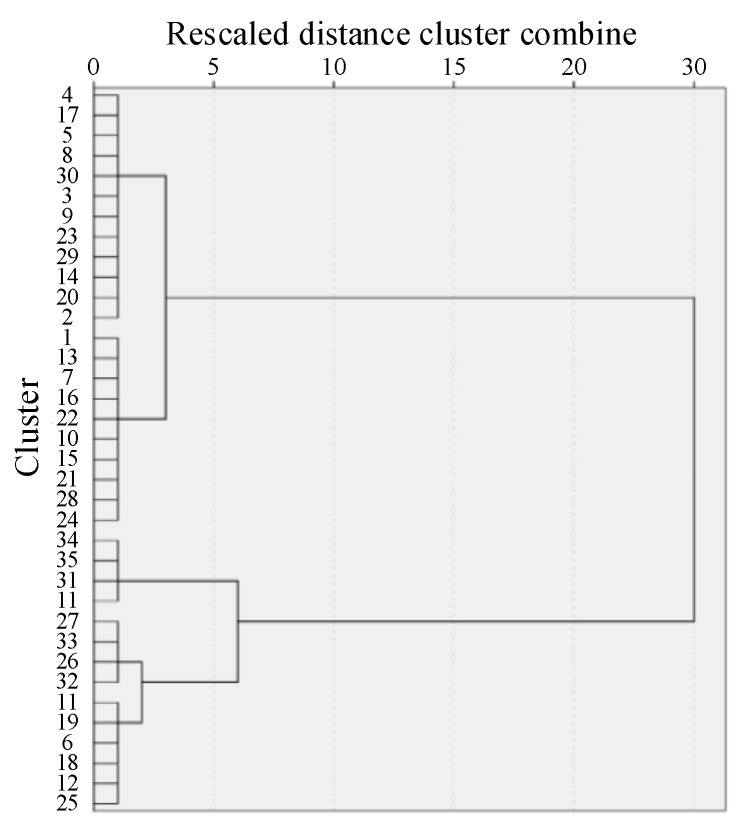
Dendrogram of clustering’s result.

The clustering analysis was done with AGNES algorithm for the sensor data. In order to find the appropriate number of classification, the dendrogram was generated, as shown in Figure 10. The clustering coefficient of the clustering process was shown in Figure 11.

**Figure 12:**
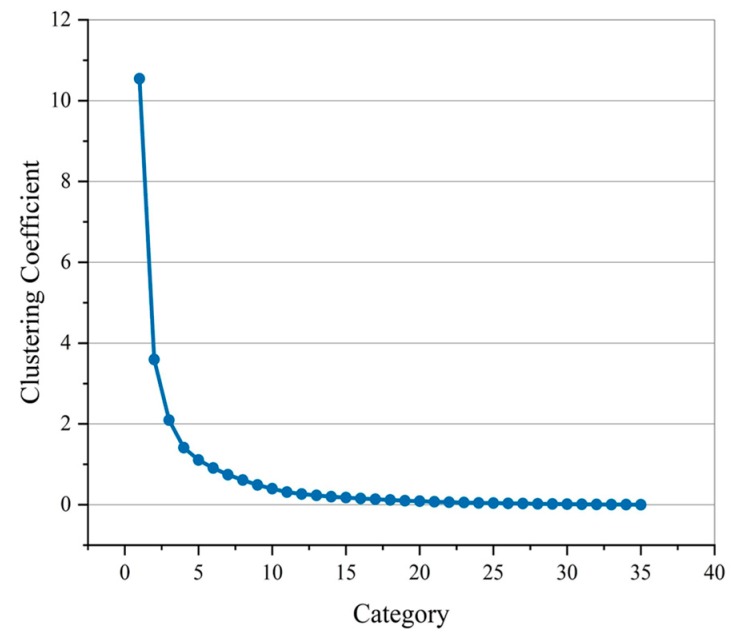
Clustering’s coefficient of different categories.

**Figure 13:**
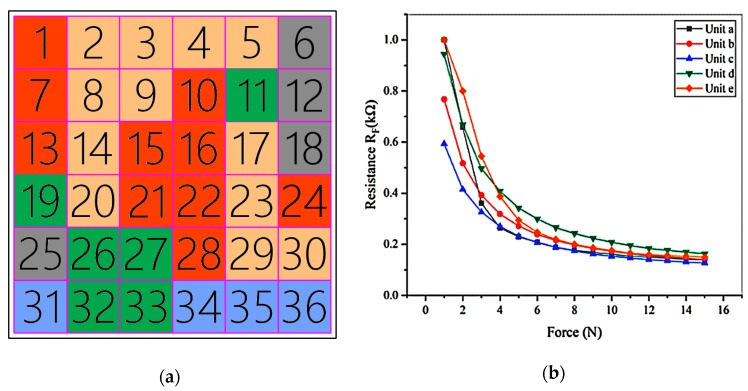
Piezoresistive fusion results: (**a**) Initial units and clustering results; (**b**) Piezoresistive curves of each unit after clustering.

As shown in Figure 11, the sensing units in different categories and the distance between categories can be seen intuitively. In Figure 12, with the number of categories increasing, the clustering coefficient decreases. When the number of categories reached about 5, the downward trend slows down. Therefore, after clustering and fusion, the sensor was merged into 5 sets from 36 initial sensing units. Similar sensing units were represented by the same color, and the piezoresistive curves of them were drawn, as shown in Figure 13a,b.

### 4.2. Force Calibration

Because the mapping between resistance and force was not obtained, a contact force detection model needs to be constructed through BP neural network training compensated samples data. The reason why the neural network is selected is that the piezoresistive characteristics of the sensor itself are nonlinear and inconsistent, and the BP neural network has good stability and robustness and is very suitable for nonlinear fitting. So BP neural network is more suitable than other algorithms.

After clustering, the sample data of each sensing units will be used for force calibration. As shown in Figure 13a,b, the sensor output resistance is non-linear, which is related to contact force and the sensing units. So the force detection model can be expressed as follows:

(14) $$R_{F}=f\left(Unit,F\right)$$

where Unit and F is the sensing unit and contact force respectively. $R_{F}$ is the sensor output resistance. To eliminate the influence of non-target parameters on the sensor output, the reverse modeling method is generally adopted. The mathematical model of force detection of the sensor in this paper can be expressed as follows:

(15) $$F=f^{-1}\left(Unit,R_{F}\right)$$

BP neural network is a multi-layer feed forward network with highly non-linear mapping ability and one-way transmission. A continuous function in any closed interval can be approximated by a BP network with an implicit layer. It is also called the universal approximation theorem [19]. In this paper, BP neural network algorithm was adopted to build the mathematical model for detecting contact force.

**Figure 14:**
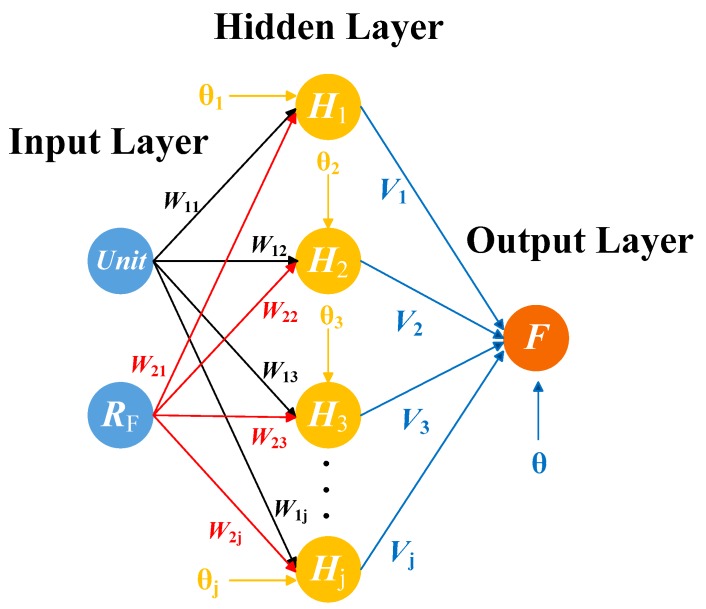
The structure of BackPropagation (BP) neural network model.

The typical structure of BP neural network contains input layer, hidden layer and output layer, as shown in Figure 14. In this paper, two neuron nodes were set in the input layer, corresponding to sensing unit and its output resistance respectively. A neuron node was set in the output layer, corresponding to contact force. The learning process is mainly composed of forward and error back propagation.

BP neural network construction process is as follows:

(1) Input samples and output samples were set:

Two neuron nodes were arranged in the input layer to correspond to sensing unit Unit at stress position and output voltage $R_{F}$. one neuron node was arranged in the output layer to correspond to the standard pressure value F.

(2) Selection of the number of hidden layer nodes:

The appropriate designing hidden layer can reduce the error and improve BP neural network training accuracy. In practice, it usually takes several experiments and a combination of artificial control to determine the final number of hidden layer nodes, and there is not absolute ideal analytic formula to calculate the number of hidden layer nodes. If the number of nodes is too little, the learning ability is limited, which is not enough to reflect all the characteristics of training samples. If the number of nodes is too many, the training time will be increased and it will reduce generalization ability, which results in overfitting. This paper adopts the following empirical formula Equation (16) to determine the approximate range of the number of nodes in the hidden layer first, and to find the best number of nodes by the method of trial.

(16) $$h=\sqrt{m+n}+a$$

where h is the number of hidden layer nodes, m is the number of input layer nodes, n is the number of nodes in the output layer, a is the adjustment constant of which the range is from 1 to 10. So, it can be calculated that the reference range of the number of hidden layer nodes in this network is 3 to 12.

(3) The connection weights $\omega_{ij}$ from the input layer to the hidden layer and the connection weights $\nu_{j}$ from the hidden layer to the output layer were initialized. And the threshold of the hidden layer neuron $\theta_{j}$ and threshold of output layer neurons θ were initialized.

(4) The hidden layer used Sigmoid as an activation function, such as Equation (17). Since the value of the Sigmoid range from 0 to 1, the values which are imported output layer needed to be scaled in any range to facilitate comparison with the values of the pressure applied to the actual calibration. Hence, the output layer used a Purelin function such as Equation (18).

(17) $$S\left(x\right)=\frac{1}{1+\exp\left(-x\right)}$$

(18) P(x)=x

The output of each unit of the hidden layer is as follows:

(19) $$\begin{array}{cc} H_{j}=S\left(\sum_{i=1}^{2}\omega_{ij}x_{i}-\theta_{j}\right) & \left(j=1,2,...,l\right) \end{array}$$

where $\omega_{ij}$ is the connection weight from the i-th node of the input layer to the j-th node of the hidden layer. $\theta_{j}$ is the threshold of the j-th neuron node of the hidden layer. l represents the number of hidden layer neuron nodes.

The output of the output layer is as follows:

(20) $$f=P\left(\sum_{j=1}^{l}\nu_{j}H_{j}-\theta \right)=\sum_{j=1}^{l}\nu_{j}H_{j}-\theta$$

where $\nu_{j}$ is the connection weight from the j node of the hidden layer to the output layer. θ is the threshold of the output layer neuron.

(5) The mean square error which is of the output values and the expected output values was calculated, as follows:

(21) $$E=\frac{1}{2}{\left(F-f\right)}^{2}$$

In order to minimize the error, the most rapid gradient descent method was adopted, and the BP network weights and thresholds were updated along the negative gradient of the error function.

The weights and threshold correction formulas for each neuron of the output layer and the hidden layer are as follows:

(22) $$\left\{ \begin{array}{l} \Delta \nu_{j}=-\eta \frac{\partial E}{\partial \nu_{j}}=\eta H_{j}\left(F-f\right)\\ \nu_{j}\left(n+1\right)=\nu_{j}\left(n\right)-\Delta \nu_{j}\left(n\right)\\ \Delta \theta =-\eta \frac{\partial E}{\partial \theta }=\eta \left(f-F\right)\\ \theta \left(n+1\right)=\theta \left(n\right)-\Delta \theta \left(n\right) \end{array}\right.$$

The weights and threshold correction formulas for the neurons in the hidden layer and the input layer are as follows:

(23) $$\left\{ \begin{array}{l} \Delta \omega_{ij}=-\eta \frac{\partial E}{\partial \omega_{ij}}=\eta \sum_{i=1}^{2}\sum_{j=1}^{l}\left(F-f\right)\nu_{j}H_{j}\left(1-H_{j}\right)x_{i}\\ \omega_{ij}\left(n+1\right)=\omega_{ij}\left(n\right)-\Delta \omega_{ij}\left(n\right)\\ \Delta \theta_{j}=-\eta \frac{\partial E}{\partial \theta_{j}}=\eta \sum_{j=1}^{l}\left(f-F\right)\nu_{j}H_{j}\left(1-H_{j}\right)\\ \theta_{j}\left(n+1\right)=\theta_{j}\left(n\right)-\Delta \theta_{j}\left(n\right) \end{array}\right.$$

where η∈(0,1) is the learning rate which controls the update step length of each iteration. n is the number of iterations.

(6) The two processes of forward propagation and error back propagation were repeated until the error reaches the set value.

Since the hidden layer of this network used Sigmoid as the activation function, the following formula should be used to normalize the sample data at first in order to improve the training speed and should be sensitivity and made the input data value fall in its saturation region which is from 0 and 1.

(24) $$\bar{Dat}\left(i\right)=\frac{Dat\left(i\right)-Dat_{\min}}{Dat_{\max}-Dat_{\min}}$$

where $\bar{Dat}\left(i\right)$ is the processed sample data, Dat(i) is the original sample data, $Dat_{\max}$ and $Dat_{\min}$ is the maximum and minimum values corresponding to the sample data respectively.

**Figure 15:**
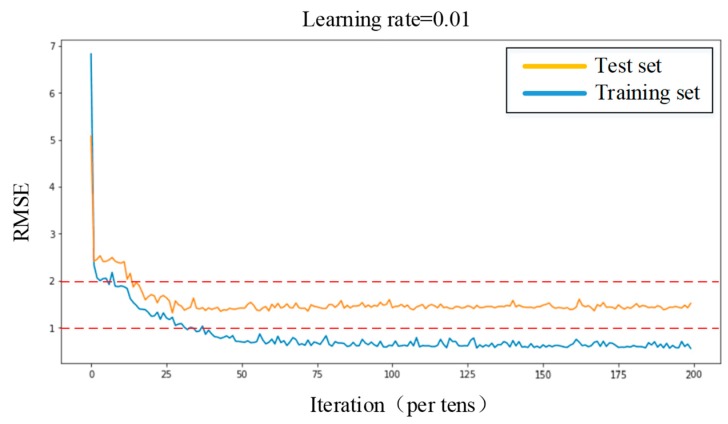
The results of BP training.

The BP neural network model was constructed by MATLAB. The output resistance of each sensing units after clustering and fusion were taken as training samples. The learning rate was set to 0.01. Adam training function combined with Mini-batch gradient descent method was used to optimize the network model and accelerate the convergence speed. The number of hidden layer nodes was adjusted to achieve the optimum training effect. After about 500 iterations, the Root Mean Squared Error (RMSE) of training set is reduced to less than 1, and the RMSE of testing set is less than 2. The final weight matrix $\left(\omega_{ij},\upsilon_{j}\right)$ and threshold matrix $\left(\theta_{j},\theta \right)$ were obtained. The results of the training were shown in Figure 15.

## 5. Experiments

### 5.1. Design and Fabrication of Sensor

The contact force detection model mentioned above needs the position information of sensing units. Based on the principle of electric field potential uniqueness, our team have already developed four-wire [20] and five-wire [21] tactile sensors in recent years. These sensors can detect the contact position by extracting the potential value distributed over the sensor film. The sensor shapes were also designed for different style, such as rectangle, sector [22] and irregular planes [23].

According to the previous research works, we improved the upper and lower electrode layers of the original structure proposed in Figure 1. Then, a novel large scale tactile sensor was designed for detecting contact position and force.

**Figure 16:**
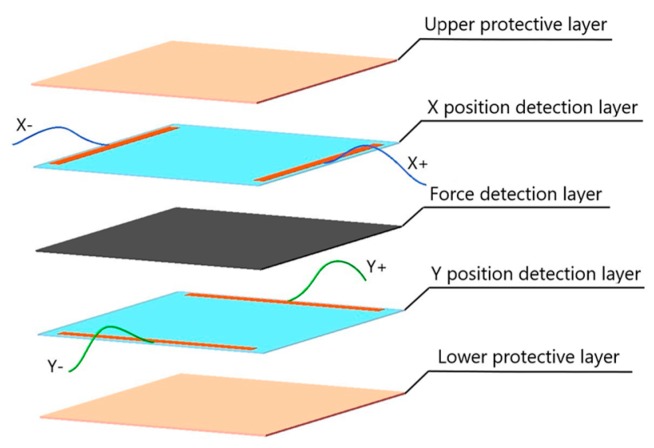
Structure of the presented sensor.

As shown in Figure 16, the position detection layer is divided into uniform electric field layers in X and Y directions. Two groups of strip line electrodes (X+, X− and Y+, Y−) are set vertically to each other on the conductive films, which are used to introduce excitation source and extract the sensor signals. The force detection layer is a piezoresistive film. The protective layers are used for collision buffering and the protection of the interior sensing materials.

**Figure 17:**
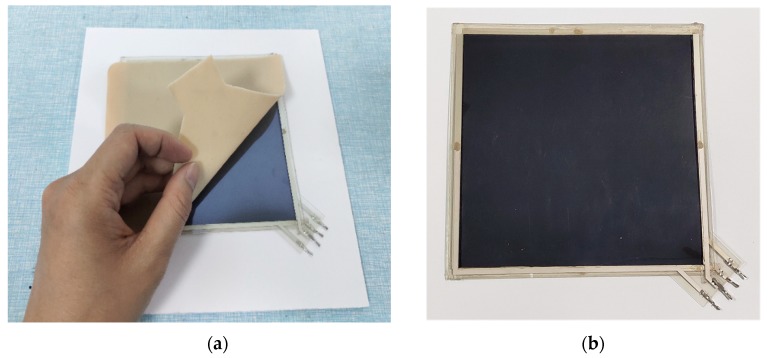
The sensor sample: (**a**)Before sensor packaging; (**b**) Sensor detection layers.

According to the electrical and physical characteristics of the tactile sensor, the sensing materials should be as light and flexible as possible. For position detection, Indium-tinoxide based on polyethylene terthalate (PET-ITO) conductive film was chosen as the layer of uniform electric field, whose thickness is only 0.125 mm. It’s uniform in conductivity, soft in flexibility and stable in chemistry. Silkscreen was adopted to print the conductive silver paste on both ends of ITO film directly as the electrodes. It can keep the sensor’s structural strength and electrical characteristics when bent. The Velostat piezoresistive film was used for the layer of force detection. A double-sided tape was used to bond the outer edge of all the layers. Finally, a dimension of 120 × 120 mm tactile sensor was successfully fabricated, as shown in Figure 17a,b.

### 5.2. the Creep and Inconsistence Test for Piezoresistive Film

**Figure 18:**
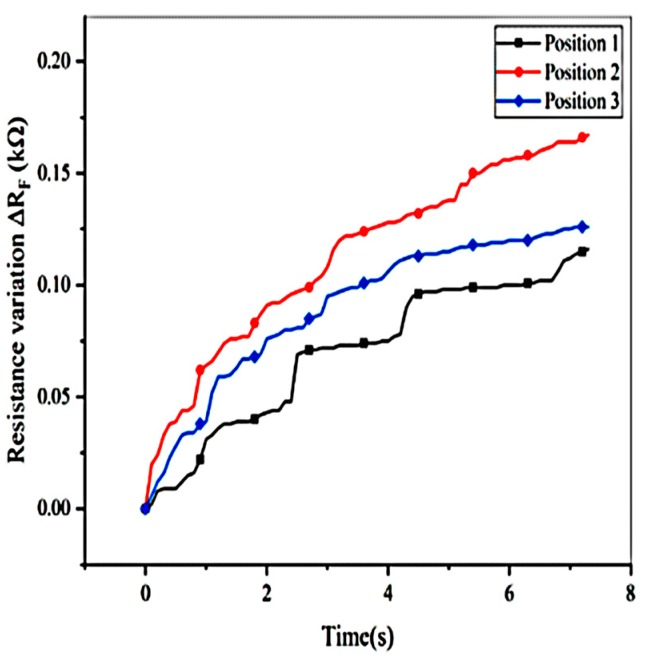
Creep at different positions under the constant force of 3 N.

Velostat is an anisotropic piezoresistive film made of polyolefins foil impregnated with carbon black, produced by the 3M Co. (Minnesota Mining and Manufacturing Company in America, Shanghai, China), limited. Its piezoresistive effect is obvious. In this paper, a 120 mm × 120 mm Velostat film was cut out to fabricate the large scale tactile sensor as a tested example. A force of 3 N by step-by-step was loaded using a compression testing machine of type ZQ-990B (Dongguan Zhihui Precision Instrument Ltd., Dongguan, China). The resistance variation caused by creep can be calculated by the formula as follows:

(25) $$\Delta R_{F}=R_{F0}-R_{F1}$$

where $R_{F0}$ and $R_{F1}$ are the sensor output resistance at initial and time t under constant force respectively. The result indicates that creeps at different positions are inconsistent under a same force, as shown in Figure 18.

**Figure 19:**
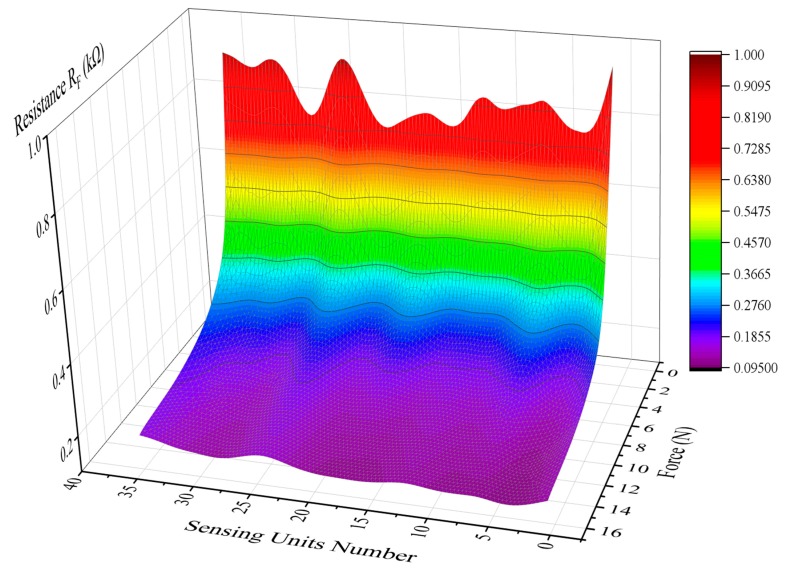
The result of surface fitting.

The sensor was divided into 36 units. The forces of 1 N to 15 N were loaded on the different units. The resistances of the sensor during loading were recorded and drawn as a three-dimensional graph. The relationship among force, units and resistance of the sensor was shown in Figure 19. It can be seen that the curve is not completely smooth and exists wrinkles. It means that the piezoresistive characteristic is inconsistent.

### 5.3. Detection Circuit

**Figure 20:**
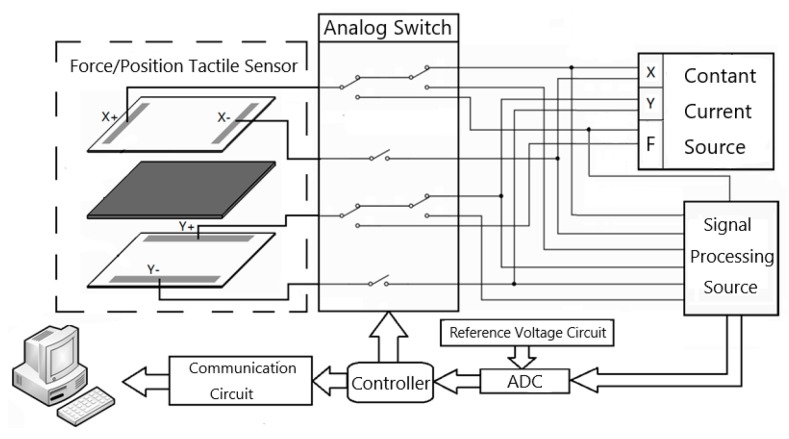
Detection system of the tactile sensor.

We built a detection circuit for the sensor, which can be powered by a 5 V battery. The whole framework is shown in Figure 20, including exciting source, analog switch, signal processing circuit, A/D conversion, reference voltage circuit, communication circuit and main controller. Among them, the exciting source consists of three constant current sources. The former two were used to constructing the uniform electric field in both X and Y directions, and the latter for contact force detection. The process of detection is as follows:

(1) The sensor’s lead wires are connected with corresponding analog switch. The controller controls the analog switch to connect different detection channels and exciting sources cyclically.
(2) The sensor’s output signals are amplified and filtered by the signal processing circuit. Then, they are input into corresponding A/D conversion ports respectively.
(3) The controller uploads the quantized data to the host computer for the algorithm calibration through the serial port. Ultimately, the contact position and force can be calculated and shown on PC.

### 5.4. Pressing Experiment after Calibration

**Figure 21:**
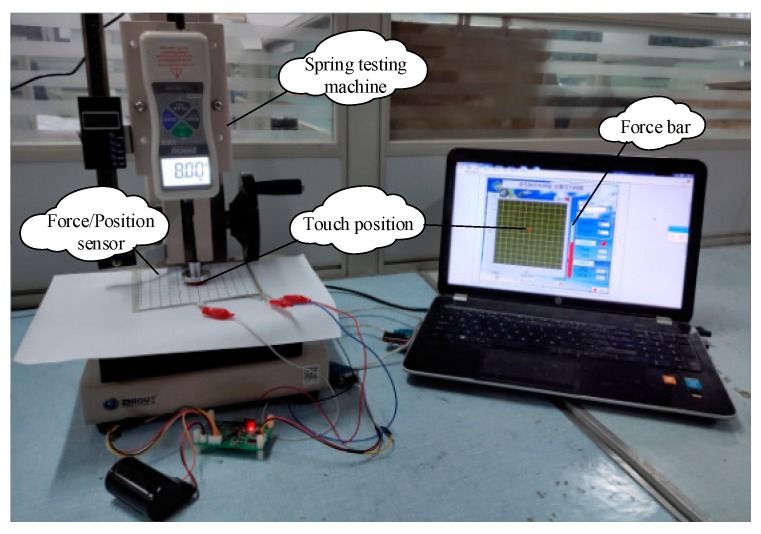
Experimental platform of compression testing machine.

In order to verify the effect of creep compensation and force calibration, the sensor was calibrated early by the methods proposed. Three pressing tests were carried out with the compression testing machine ZQ-20B-1 (Dongguan Zhihui Precision Instrument Ltd., Dongguan, China), and the experimental platform is shown in Figure 21.

**Figure 22:**
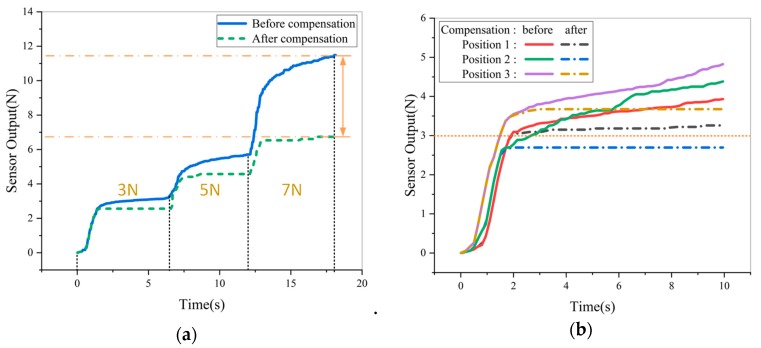
Sensor output before and after creep compensation: (**a**) Imposing force incremental step by step; (**b**) Different positions.

Firstly, an incremental stepper force was applied on it and kept for 5 s after each increase. Then, a force of 3 N was applied to three positions of the sensor sample in turn. The results were shown in Figure 22a,b.

As shown in Figure 22, the results of force detection are quite different between before creep compensation and after creep compensation. Without compensation, the maximum error is up to 5 N after loading to 7 N. The error will continue to increase as time goes on. By contrast, the compensated sensor output is more stable, which can provide more accurate feedback information of force. Above all, the effect of compensation is not affected by the pressed position and force basically. It extends application range of the sensor, such as stabilizing grasping operation of dexterous hands and plantar pressure distribution testing.

**Figure 23:**
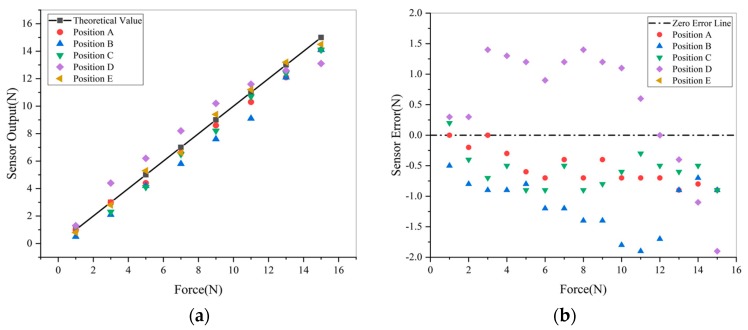
The results of force contact experiment: (**a**) The result of contact force detection; (**b**) Error distribution of contact force detection.

Finally, four corners (A, B, C, D) and center (E) of the sensor sample were selected as the contact positions, and a force of 1 N to 15 N was applied to them individually. As shown in Figure 23a,b, the maximum error of force detection is less than 2 N, and most of the error can be maintained within 1 N.

## 6. Conclusions

Based on K-means clustering and fuzzy recognition, a creep tracking compensation method was proposed in this paper for creep inconsistency of large scale piezoresistive film. It guarantees the data collected for calibration more accurate and stable. Then, the sensor was calibrated for piezoresistive inconsistency and nonlinearity of the large scale tactile sensor. Firstly, different sensing units were classified and fused by hierarchical clustering algorithm. The fusion can reduce the complexity of subsequent neural network fitting and reduce boundaries between different sensing units, and prevent overfitting. Secondly, introducing the position information of sensing units, the BP neural network was used to build the contact force detection model. The BP neural network can solve problem which is the nonlinear and inconsistent characteristics of the piezoresistive sensor. Finally, the sensor sample was fabricated, and the experiments were carried out by the calibrated sensor detection system. The results demonstrate that the methods proposed can compensate the creep effectively. Besides, the computation in large-scale sensor calibration is reduced to a certain extent by decreasing redundant calibration data through the hierarchical clustering. In summary, these methods are valuable for designing large-scale piezoresistive tactile sensor with force detection.
